# Chinese thyroid imaging reporting and data system with redefined marked hypoechogenicity for thyroid malignancy risk stratification demonstrates improved diagnostic accuracy

**DOI:** 10.7717/peerj.20817

**Published:** 2026-02-11

**Authors:** Yingxue Bai, Mingxin Yu, Size Wu

**Affiliations:** Department of Ultrasound, The First Affiliated Hospital of Hainan Medical University, Haikou, Hainan Province, China

**Keywords:** Thyroid, Malignancy, Ultrasound, Thyroid nodule, Thyroid imaging reporting and data system, Stratification, Chinese

## Abstract

**Purpose:**

Using ultrasound to distinguish between benign and malignant thyroid nodules can be challenging. The purpose of this study was to evaluate the diagnostic performance of a modified Chinese Thyroid Imaging Reporting and Data System (C-TIRADS) that incorporates a redefined criterion for marked hypoechogenicity in stratifying the malignancy risk of thyroid nodules.

**Methods:**

In this retrospective study, we analyzed patients with thyroid nodules who underwent ultrasound examination and subsequent biopsy or treatment at a tertiary hospital between January 2022 and December 2023. Interobserver agreement in identifying marked hypoechogenicity between two reviewers was assessed using kappa statistics. Using histopathology as the reference standard, the diagnostic performance of three classification systems, including the classical C-TIRADS, the modified C-TIRADS, and the 2021 K-TIRADS, was compared in terms of sensitivity, specificity, positive predictive value (PPV), negative predictive value (NPV), accuracy, and the area under the receiver operating characteristic curve (AUC).

**Results:**

The study included 1,219 patients with 1,721 thyroid nodules. After redefining marked hypoechogenicity, interobserver agreement (kappa) improved from 0.652 to 0.722. Compared to the original criterion, the redefined marked hypoechogenicity demonstrated significantly higher sensitivity (74.5% *vs.* 15.1%), accuracy (86.8% *vs.* 70.8%), and AUC (0.836 *vs.* 0.563) (all *p* < 0.05), with only a marginal reduction in specificity (92.6% *vs.* 97.4%, *p* > 0.05). The malignancy rates for the modified C-TIRADS categories were as follows: 12.0% (4a), 60.6% (4b), 85.8% (4c), and 93.3% (5). The optimal diagnostic thresholds were category 4b for both classical and modified C-TIRADS and category 4 for K-TIRADS. The modified C-TIRADS achieved superior accuracy (90.2% *vs.* 88.2% *vs.* 81.3%) and AUC (0.908 *vs.* 0.870 *vs.* 0.857) compared to the classical C-TIRADS and K-TIRADS, respectively (all pairwise *p* < 0.05).

**Conclusion:**

The modified C-TIRADS, incorporating a redefined criterion of marked hypoechogenicity, shows improved diagnostic performance in stratifying the malignancy risk of thyroid nodules and provides risk assessments that align more closely with the expected probabilities outlined in the (classical) C-TIRADS guidelines.

## Introduction

Thyroid nodules are a common clinical finding in the adult population, encompassing a spectrum of lesions ranging from benign to malignant (Grani et al., 2024). Globally, thyroid cancer accounted for 4.1% of all new cancer cases in 2022, with a mortality rate of 0.5% (Bray et al., 2024). Early detection and appropriate management of thyroid cancer are crucial for improving patient prognosis and quality of life. Ultrasound plays a central role in the initial detection of thyroid nodules; however, accurately differentiating between benign and malignant features remains challenging. Fine-needle aspiration (FNA) is widely used for definitive diagnosis (Alexander et al., 2020; Russ, Trimboli & Buffet, 2021; Kim et al., 2023). To standardize ultrasound reporting, optimize malignancy risk stratification, reduce unnecessary FNA procedures, and guide clinical decision-making, the Thyroid Imaging, Reporting and Data System (TIRADS) was introduced, building on the systematic analysis of key sonographic features (Horvath et al., 2009; Russ, Trimboli & Buffet, 2021; Kim et al., 2023; Yang et al., 2022). Among the various TIRADS versions developed worldwide, the Chilean TIRADS (2009) represents the first such system, while the (classical) Chinese TIRADS 2020 (Chinese Thyroid Imaging Reporting and Data System, C-TIRADS) is among the most recent (Zhou et al., 2020). Although existing TIRADS frameworks demonstrate strong risk stratification capabilities, their clinical adoption varies considerably due to differences in practicality and diagnostic performance (Russ, Trimboli & Buffet, 2021; Kim et al., 2023; Yang et al., 2022; Zhou et al., 2020). For example, the pioneering TIRADS proposed by Horvath et al. (2009) was not widely adopted due to its complexity, which motivated subsequent revisions aimed at improving usability (Ha et al., 2021). Similarly, the 2021 Korean TIRADS (K-TIRADS) refined earlier versions from 2011 and 2016, showing enhanced diagnostic accuracy in evaluating thyroid malignancies (Ha et al., 2021). In light of these ongoing developments, recent research has increasingly focused on comparing the efficacy, usability, and potential refinements of different TIRADS (Russ, Trimboli & Buffet, 2021; Kim et al., 2023; Nabahati & Moazezi, 2022; Lu et al., 2023). Classical C-TIRADS employs a scoring system based on malignancy-associated ultrasound features, where a higher score indicates an increased risk of cancer (Yang et al., 2022; Zhou et al., 2021; Chen, Lin & Wu, 2022; Topcuoglu et al., 2025; Qi et al., 2021; Cai et al., 2023a; Cai et al., 2023b; Si et al., 2024). While praised for its clinical practicality, classical C-TIRADS has been noted to exhibit an imbalance between sensitivity and specificity, particularly within the higher-risk categories 4c and 5 (Chen, Lin & Wu, 2022; Topcuoglu et al., 2025; Si et al., 2024). This is reflected in the considerable variation reported for the malignancy rate of category five nodules. For instance, studies have reported rates ranging from 60.86% to 85.29% in certain cohorts (Chen, Lin & Wu, 2022; Qi et al., 2021), while others have observed rates as high as 93.5% to 100% (Qi et al., 2021; Cai et al., 2023a; Cai et al., 2023b; Si et al., 2024). It is noteworthy, however, that in the latter studies, these category five nodules constituted only a small proportion (1.96% to 6.8%) of all confirmed malignant cases.

The degree of hypoechogenicity in thyroid nodules is recognized as a significant predictor of malignancy. As such, its precise definition and accurate identification are essential for appropriate TIRADS categorization and optimal diagnostic performance (Lee et al., 2020; Lee et al., 2022; Delfim et al., 2023; Hu et al., 2024). However, markedly hypoechoic malignant nodules account for less than 40% of all malignant thyroid nodules presenting with any degree of hypoechogenicity (Lee et al., 2020; Lee et al., 2022; Delfim et al., 2023). According to (classical) C-TIRADS 2020, marked hypoechogenicity is defined as echogenicity lower than that of the adjacent strap muscle (Zhou et al., 2020). Yet, current evidence indicates that only 13.9–28.03% of malignant nodules fulfill this criterion across different studies (Liu et al., 2023; Chen, Lin & Wu, 2022; Cai et al., 2023a; Cai et al., 2023b; Liu et al., 2023), suggesting that the current definition may be suboptimal for effective malignancy risk stratification. In response to this limitation, Liu et al. (2023) proposed a revised definition of marked hypoechogenicity, characterizing it as echogenicity lower than or equal to that of the adjacent strap muscle. This redefined criterion has shown improved interobserver agreement (κ = 0.722) and enhanced diagnostic performance. Nevertheless, given the inherent subjectivity in ultrasound interpretation (De Carlos et al., 2024), further validation is warranted. Therefore, the objective of this retrospective study was to evaluate the diagnostic performance of a modified C-TIRADS that incorporates the redefined markedly hypoechoic criterion, and to compare it with the classical C-TIRADS 2020 and K-TIRADS 2021.

## Materials & Methods

### Study population selection

**Figure 1:**
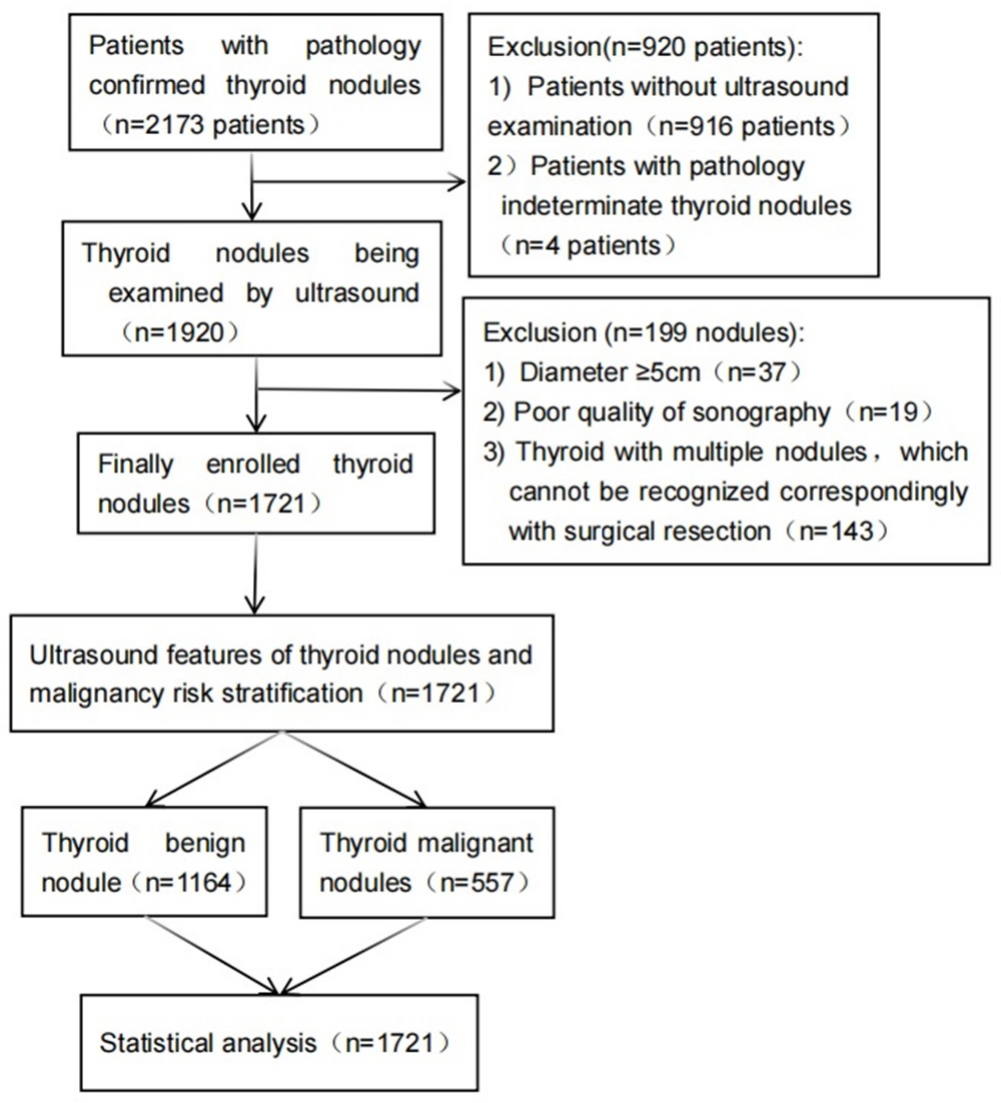
Flowchart of study sample selection and analysis.

This retrospective study was approved by the Ethics Committee of The First Affiliated Hospital of Hainan Medical University (Approval No. 2024-KYL-021), and written informed consent was obtained form all patients. We reviewed ultrasound images and clinical data from patients who underwent surgical resection or core needle biopsy for thyroid nodules at The First Affiliated Hospital of Hainan Medical University between January 2020 and December 2023. The collected data included demographic information (age, sex), relevant medical history, and definitive histopathological diagnoses. The inclusion criteria were as follows: (1) availability of thyroid ultrasound images with diagnostic quality; (2) a time interval of fewer than two months between the ultrasound examination and histopathological analysis; (3) for patients with multiple nodules with different suspicion levels of malignancy, the single most suspicious nodule of malignancy was selected; (4) for patients with multiple benign-appearing nodules and with similar suspicion levels, the nodule with the largest diameter was prioritized. The exclusion criteria included: (1) poor image resolution or incomplete views (missing transverse or longitudinal planes); (2) indeterminate histopathological or immunohistochemical results; (3) coexistence of benign and malignant nodules within the same thyroid lobe with ambiguous imaging boundaries; (4) nodules with a maximum diameter of ≥5 cm; (5) spongiform nodules or lesions that were predominantly cystic. A detailed flowchart of the patient selection process is presented in Fig. 1.

### Ultrasound examination

Thyroid ultrasound examinations were performed using seven different ultrasound systems: the Mindray DC 8, Mindray Resona 7, and Mindray Eagus R9S (Shenzhen Mindray Bio-Medical Electronics Co., Ltd., Shenzhen, China); the Aloka Prosound α-10 (Hitachi Aloka Medical Ltd, Tokyo, Japan); the GE Logiq 9 and GE Logiq E9 (General Electric Healthcare); and the Philips EPIQ5 (Philips Healthcare, Amsterdam, Netherlands). All systems were equipped with high-frequency linear transducers (range: 5–15 MHz). The examinations were conducted by a total of 12 radiologists, each with 2 to 29 years of experience in thyroid imaging. During the procedure, patients were placed in the supine position with the neck fully exposed. Adequate coupling gel was applied to the anterior cervical region. Systematic scanning was performed in both transverse and longitudinal planes to comprehensively assess the thyroid isthmus and bilateral lobes. Throughout image acquisition, minimal transducer pressure was applied to the skin to avoid structural distortion of the thyroid tissue. When a thyroid nodule was identified, its location, size, and key sonographic features were systematically documented. These features included shape, margin characteristics, echogenicity (including solid/cystic composition and internal echoes), vertical orientation (taller-than-wide), the presence of microcalcifications, evidence of extrathyroidal extension, posterior acoustic features, vascularity patterns, and the status of cervical lymph nodes (if abnormal or enlarged). Representative images of a thyroid nodule were saved in Picture Archiving and Communication System (PACS).

### Overview of (classical) C-TIRADS 2020

The (classical) C-TIRADS (2020) stratifies the malignancy risk of thyroid nodules based on a weighted scoring system of specific ultrasound features (Zhou et al., 2020). The system assigns points as follows: Malignancy-suspicious features (one point each): solid composition, the presence of microcalcifications, vertical orientation (taller-than-wide), marked hypoechogenicity (defined as echogenicity lower than that of the adjacent cervical strap muscle, which serves as the reference), and ill-defined or irregular margins or extrathyroidal extension. Benign indicator (−1 point): the presence of a comet-tail artifact. The final C-TIRADS category is determined by the aggregate score: Category 1: Normal thyroid gland (no nodules present). Category 2: Total score = −1 (benign). Category 3: Total score = 0 (low risk). Category 4a: Total score = 1 (low-intermediate risk). Category 4b: Total score = 2 (intermediate risk). Category 4c: Total score = 3–4 (high risk). Category 5: Total score ≥ 5 (very high risk). Category 6: Biopsy-proven malignancy.

### Overview of modified C-TIRADS

The modified C-TIRADS retains the original scoring framework of the C-TIRADS 2020 but incorporates a redefined criterion of marked hypoechogenicity, changing it from “echogenicity lower than that of the adjacent cervical strap muscle” to “echogenicity lower than or equal to that of the adjacent cervical strap muscle” (Liu et al., 2023). All other scoring criteria and category thresholds remain unchanged.

### Overview of K-TIRADS

The K-TIRADS 2021 version stratifies nodules based on their composition, echogenicity, and the presence of suspicious features (Ha et al., 2021). The suspicious features include punctate echogenic foci (microcalcifications), nonparallel orientation (taller-than-wide), and irregular margins. The categories are defined as follows: Category 1: No nodule. Category 2: Benign patterns (*e.g.*, spongiform, or purely cystic with comet-tail artifacts). Category 3: Partially cystic or iso-hyperechoic nodule without any suspicious features. Category 4: A solid hypoechoic nodule without suspicious features; OR a partially cystic/iso-hyperechoic nodule with one or more suspicious features; OR an entirely calcified nodule. Category 5: A solid hypoechoic nodule with one or more suspicious features.

### Ultrasound feature analysis and TIRADS categorization

A research assistant, who did not participate in subsequent image interpretation, retrieved all ultrasound images and corresponding histopathology reports from the PACS. Relevant medical history and laboratory results were obtained from the Hospital Information System. For patients who underwent multiple ultrasound examinations, the examination closest to the date of histopathological confirmation (within two months) was selected for analysis. Two radiologists specializing in thyroid ultrasound, with 2 and 20 years of experience respectively, independently evaluated the image quality, including resolution and completeness of transverse and longitudinal views, and assessed all eligible thyroid nodules. Each nodule was classified according to the C-TIRADS 2020, the modified C-TIRADS, and the K-TIRADS criteria. Any discrepancies in assessment were resolved through a consensus discussion between the two radiologists.

The histopathological results from surgical resection or core needle biopsy served as the reference standard for diagnosis. Both radiologists were blinded to the final histopathological results and any prior ultrasound reports throughout the image evaluation process.

### Statistical analysis

Continuous variables following a normal distribution are presented as mean ± standard deviation and were compared using the independent Student’s *t*-test. Non-normally distributed continuous variables are expressed as median with interquartile range (IQR) and were compared using the Mann–Whitney U test. Categorical variables are summarized as counts and percentages and were analyzed using Pearson’s chi-square test. To evaluate interobserver agreement, a random subset of 40 thyroid nodules (one per patient) was selected. The two radiologists independently assessed these nodules using both the original and redefined markedly hypoechoic criteria, as well as for classical and modified C-TIRADS categorizations. Agreement was quantified using Cohen’s kappa (κ), interpreted as follows (Landis & Koch, 1977): 0.00–0.20, poor; 0.21–0.40, fair; 0.41–0.60, moderate; 0.61–0.80, substantial; and 0.81–1.00, almost perfect. The sensitivity, specificity, positive predictive value (PPV), negative predictive value (NPV), accuracy, and area under the receiver operating characteristic (ROC) curve (AUC) were calculated for both the original and redefined markedly hypoechoic criteria. Differences in sensitivity and specificity between the two criteria were compared using McNemar’s test. The optimal diagnostic cutoff values for classical C-TIRADS, modified C-TIRADS, and K-TIRADS were determined by maximum Youden index. Comparisons of the AUC values among the different TIRADS versions were performed using DeLong’s test. A two-tailed *p*-value of less than 0.05 was considered statistically significant. All statistical analyses were performed using SPSS version 25.0 (IBM Corp., Armonk, NY, USA) and MedCalc version 23.7 (MedCalc Software Ltd, Ostend, Belgium).

## Results

### Clinical characteristics and pathological distribution

A total of 1,219 patients with 1,721 thyroid nodules were included in the final analysis. The patient enrollment process, detailed in Fig. 1, led to the exclusion of 920 patients whose thyroid nodules lacked corresponding ultrasound examinations and four patients with indeterminate histopathological results. The study cohort comprised 1,219 patients, with a mean age range of 13 to 84 years. The population included 231 (18.9%) males and 988 (81.1%) females. Among the 1,721 analyzed nodules, 1,164 were benign and 557 were malignant, yielding a benign-to-malignant ratio of approximately 2:1. The mean diameter of benign nodules (20.35 ± 12.17 mm; range: 4.2–50.0 mm) was significantly larger than that of malignant nodules (11.75 ±8.17 mm; range: 4.7–49.0 mm). The detailed distribution of pathological types is summarized in Table 1.

**Table 1:** Histopathologies of the thyroid nodules.

Benign thyroid nodule	Number	Malignant thyroid nodule		Number
		Papillary carcinoma		
Nodular goiter	750		Classical variant	479
Follicular nodule	104		Follicular variant	20
Chronic thyroiditis	99		Solid variant	3
Follicualr adenoma	71		Diffuse sclerosing variant	2
Toxic nodular goiter	20		Tall cell variant	1
Subacute thyroiditis	6		Clear cell variant	1
Eosinophilic adenoma	7		Papillary carcinoma with unknown variant	42
Colloid cyst	1	Follicular adenocarcinoma		6
Benign lesion with unknown variant	106	Medullar carcinoma		2
		Metastasis of chondrosarcoma (Biopsy)		1
Total	1,164	Total		557

### Diagnostic performance of ultrasound features and modified C-TIRADS

**Figure 2:**
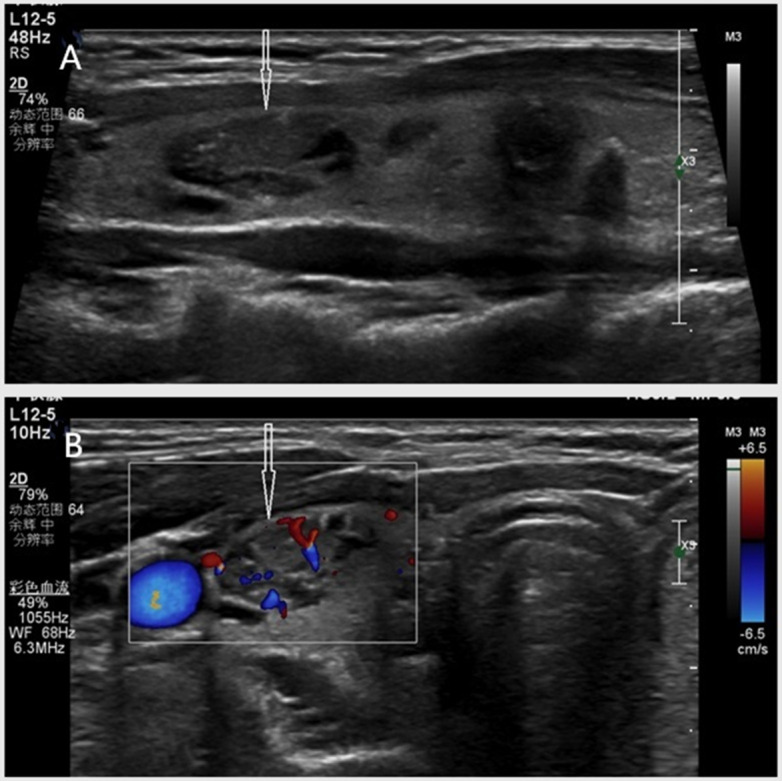
(A–B) A 45-year-old female with a pathologically confirmed thyroid nodule (follicular hyperplasia). Ultrasound demonstrates a hypoechoic nodule (arrows indicate) in the right thyroid lobe with the following features: elliptical shape (no taller-than-wide), mixed solid-cystic composition, scattered punctate hyperechoic foci, well-defined margins, and dimensions of 16.8 × 9.9 × 8.2 mm. No posterior acoustic features observed. Classification: classical C-TIRADS 4a, modified C-TIRADS 4a, K-TIRADS 4.

**Figure 3:**
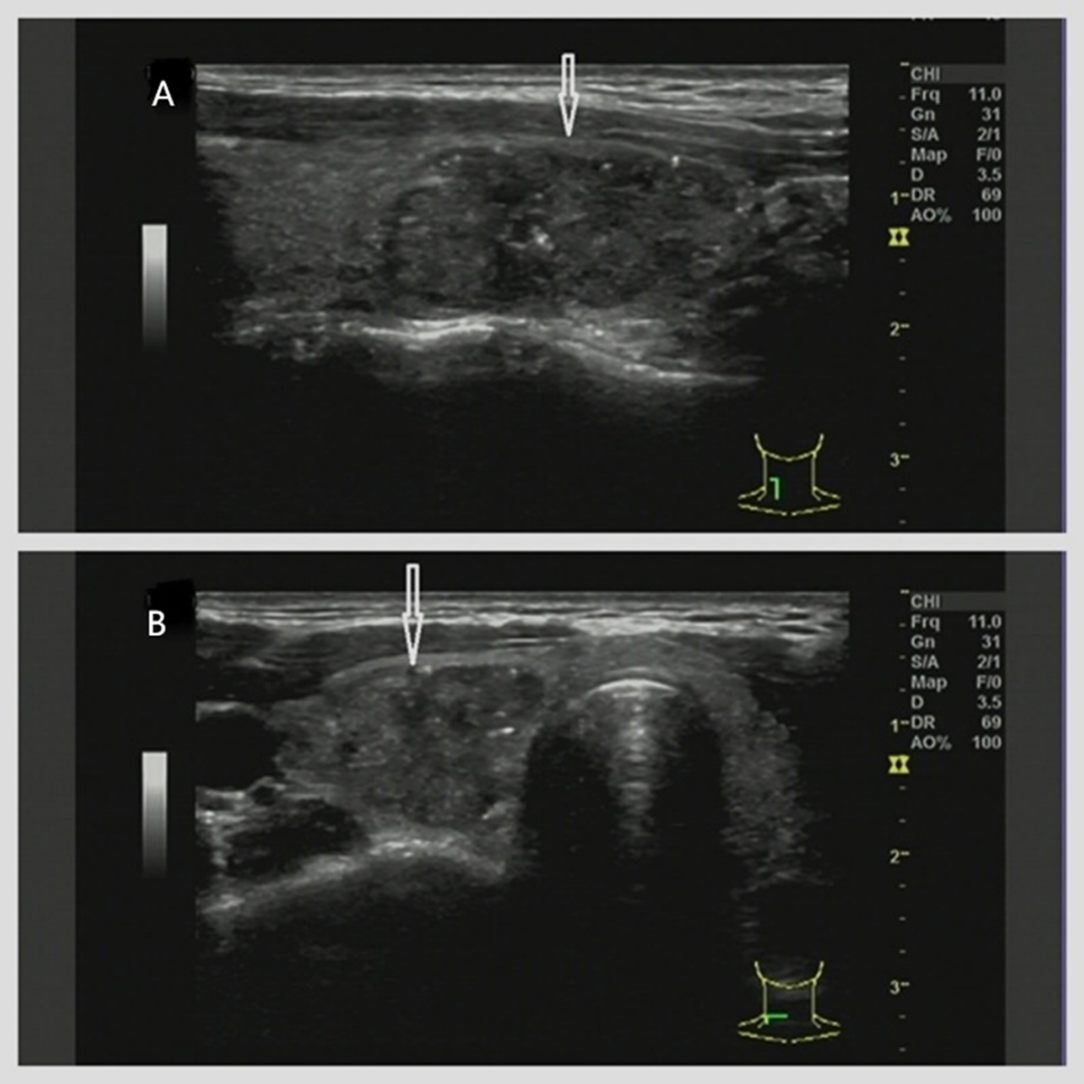
(A–B) A 29-year-old female with a pathologically confirmed thyroid nodule (papillary carcinoma). Ultrasound reveals a hypoechoic nodule (arrows indicate) in the right thyroid lobe with the following characteristics: irregular shape (no taller-than-wide), mixed solid-cystic composition, scattered punctate hyperechoic foci, comet-tail artifact, partially discernible margins, and dimensions of 27.7 × 19.1 × 13.0 mm. No posterior acoustic features observed. Classification: classical C-TIRADS 4b, modified C-TIRADS 4c, K-TIRADS 4.

**Figure 4:**
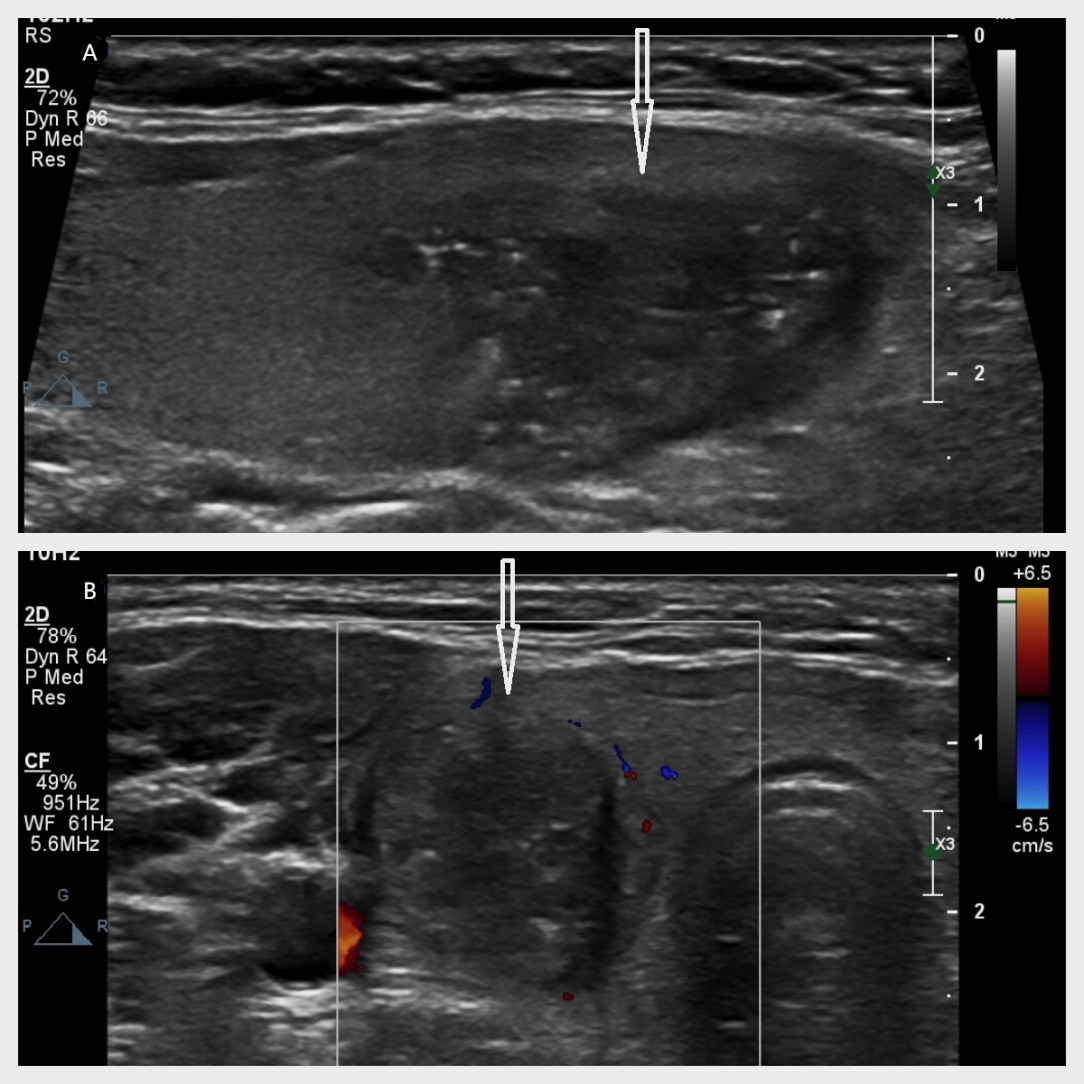
(A–B) A 30-year-old female with a pathologically confirmed thyroid nodule (papillary carcinoma). Ultrasound shows a hypoechoic nodule (arrows indicate) in the right thyroid lobe with the following features: irregular shape (no taller-than-wide), entirely solid composition, scattered punctate hyperechoic foci, partially discernible margins, and dimensions of 30.5 × 20.8 × 14.4 mm. No posterior acoustic features observed. Classification: classical C-TIRADS 4c, modified C-TIRADS 4c, K-TIRADS 5.

**Figure 5:**
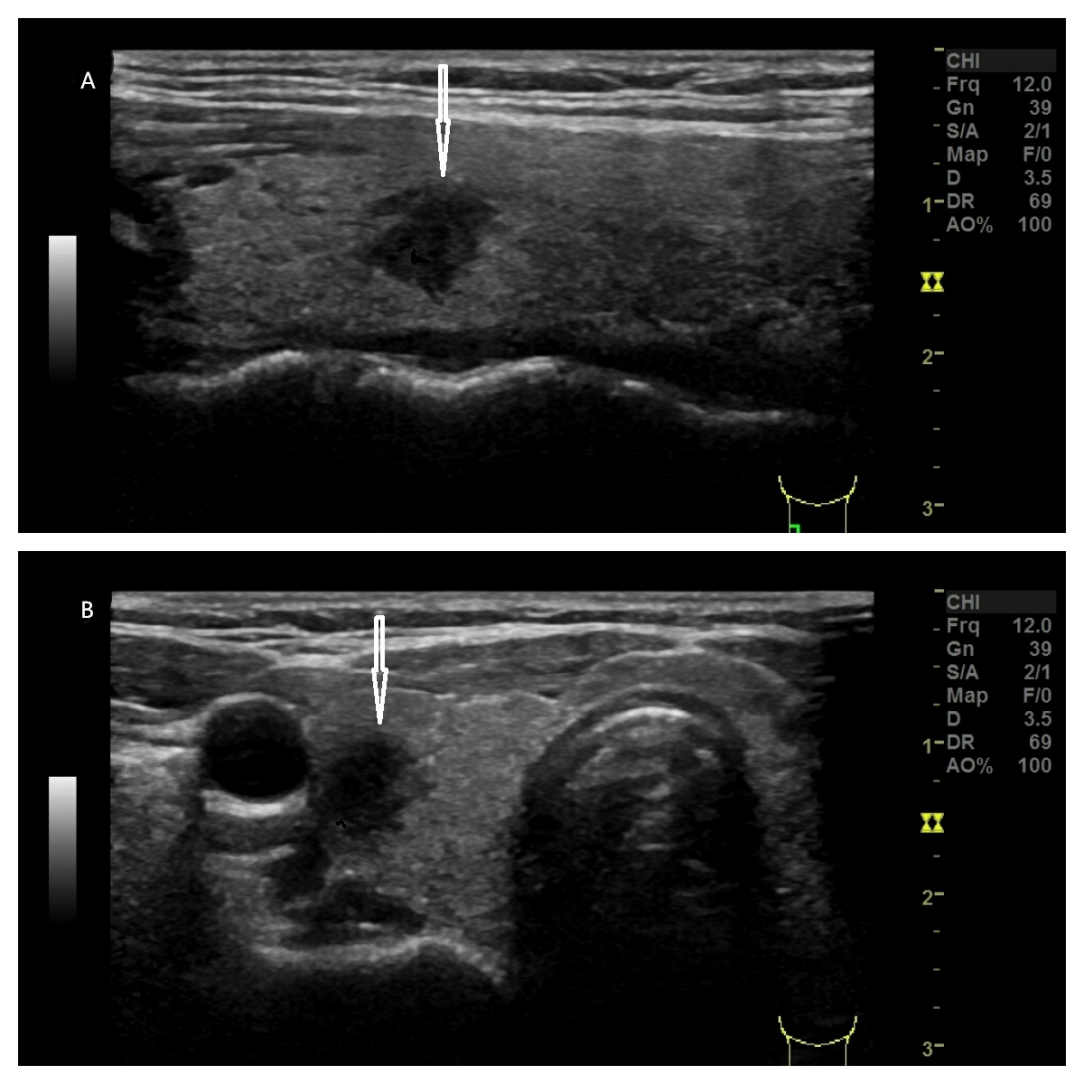
(A–B) A 37-year-old female with a pathologically confirmed thyroid nodule (papillary carcinoma). Ultrasound demonstrates a markedly hypoechoic nodule (arrows indicate) in the right thyroid lobe with the following characteristics: irregular shape (with taller-than-wide), entirely solid composition, two fine punctate hyperechoic foci, partially discernible margins, and dimensions of 8.1 × 6.2 × 7.4 mm. No posterior acoustic features observed. Classification: classical C-TIRADS 5, modified C-TIRADS 5, K-TIRADS 5.

**Figure 6:**
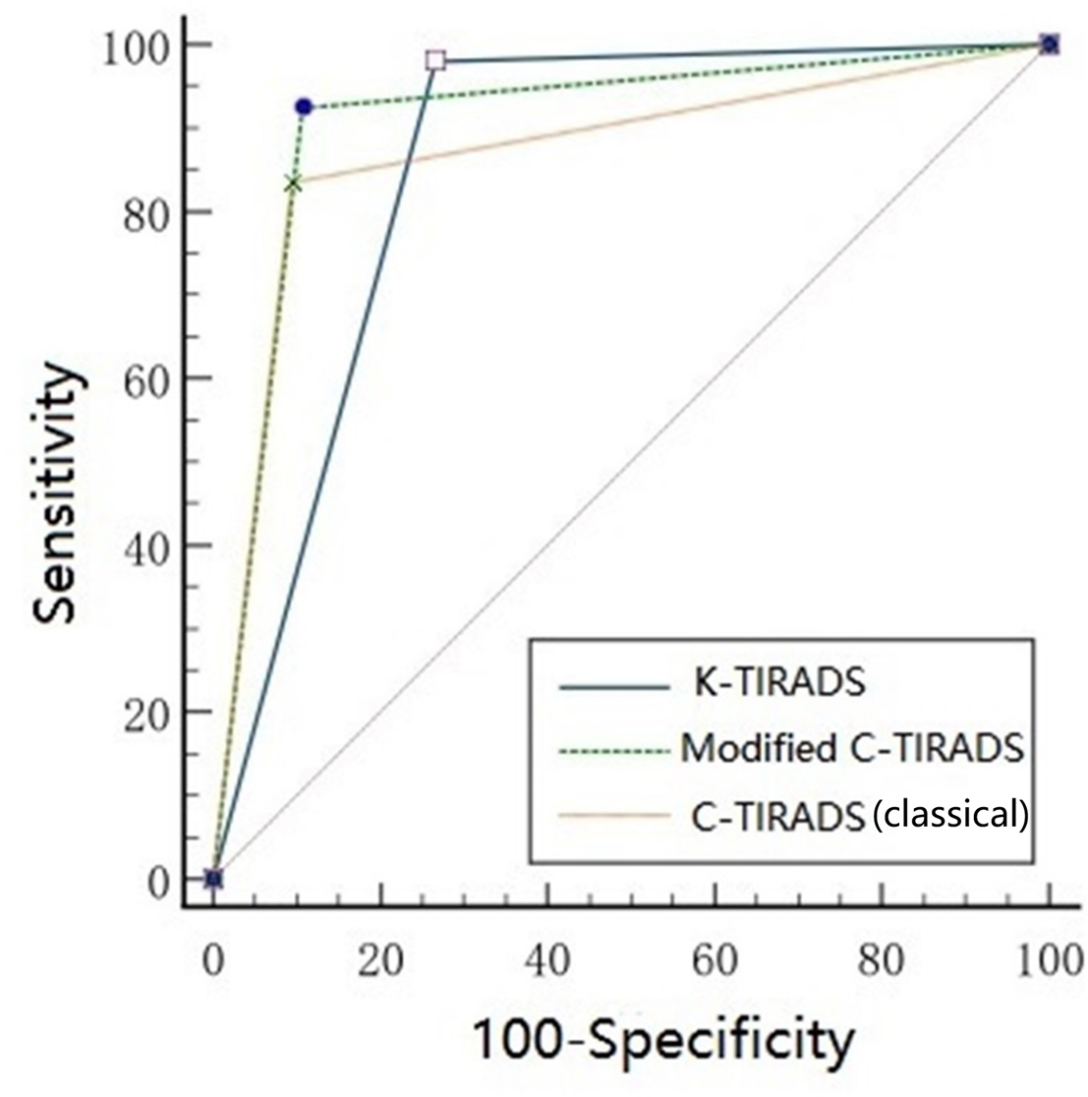
Comparative performance of classical C-TIRADS, modified C-TIRADS, and K-TIRADS in malignancy risk stratification for thyroid nodules.

Ultrasound features associated with a high likelihood of malignancy demonstrated a statistically significant(*p* < 0.001 for all), including vertical orientation, solid composition, marked hypoechogenicity, microcalcifications, and ill-defined/irregular margins or extrathyroidal extension. Comet-tail artifact associated with benignity of the thyroid nodule was observed in 35 of the 1,164 benign nodules but in none of the 557 malignant nodules (*p* < 0.001). The redefined criterion for marked hypoechogenicity yielded significantly improved diagnostic performance compared to the classical definition. Specifically, it showed markedly higher sensitivity (74.5% *vs.* 15.1%) and a greater area under the ROC curve (AUC: 0.836 *vs.* 0.563), with only a modest reduction in specificity (92.6% *vs.* 97.4%). Overall accuracy also increased from 70.8% to 86.8%, with all differences being statistically significant (*p* < 0.001). Detailed results are provided in Table 2. Interobserver agreement for identifying markedly hypoechoic and hypoechoic features, as well as for classical and modified C-TIRADS categorizations, is summarized in Table 3. When applying the classical and modified C-TIRADS for malignancy risk stratification, the observed malignancy rates in categories 2, 3, and 4c aligned with those suggested in the (classical) C-TIRADS 2020 guidelines. Rates in categories 4a and 4b were higher than guideline suggestions. Notably, the malignancy rate in category 5 under the classical C-TIRADS was lower than the guideline reference, whereas the rate under the modified C-TIRADS closely matched the expected value. These findings are detailed in Table 4. Representative ultrasound images and corresponding categorizations according to classical C-TIRADS, modified C-TIRADS, and K-TIRADS are illustrated in Figs. 2 to 5. Based on the maximum Youden index, the optimal diagnostic cutoff for malignancy risk stratification was category ≥4b for both classical and modified C-TIRADS, and category ≥4 for K-TIRADS. At these cutoffs, the sensitivity and specificity were as follows: classical C-TIRADS (sensitivity: 83.1%, specificity: 90.5%), modified C-TIRADS (sensitivity: 89.5%, specificity: 89.2%), and K-TIRADS (sensitivity: 90.0%, specificity: 73.4%). Complete results are summarized in Table 5. Further diagnostic performance metrics (sensitivity, specificity, PPV, NPV, accuracy, and AUC) for each TIRADS version at their respective optimal cutoffs are provided in Table 6. In pairwise comparisons, modified C-TIRADS achieved the highest accuracy, classical C-TIRADS showed the highest specificity, and K-TIRADS exhibited the highest sensitivity but the lowest specificity and accuracy. All pairwise differences in sensitivity, specificity, and accuracy were statistically significant (*p* < 0.001 for all). The AUC values for K-TIRADS, classical C-TIRADS, and modified C-TIRADS increased sequentially. Modified C-TIRADS demonstrated a significantly higher AUC than both classical C-TIRADS and K-TIRADS (*p* < 0.001 for both), while no significant difference was observed between K-TIRADS and classical C-TIRADS (*p* = 0.169), as shown in Fig. 6.

**Table 2:** Diagnostic efficacy of ultrasound features of high likehood of malignancy.

	Sensitivity (%)	Specificity (%)	Accuracy (%)	AUC (95% CI)
Vertical orientation	48.1	92.6	78.2	0.704 (0.675–0.732)
Solid composition	71.3	85.2	80.7	0.782 (0.762–0.802)
Microcalcifications	57.6	88.6	78.6	0.731 (0.709–0.752)
Ill-defined/irregular margin or extrathyroidal extension	75.9	89.2	84.9	0.826 (0.807–0.843)
Markedly hypoechoic (Classical)	15.1	97.4	70.8	0.563 (0.539–0.586)
Markedly hypoechoic(Redefined)	74.5	92.6	86.8	0.836 (0.817–0.853)

**Notes.**

Note AUC Area under the receiver operating characteristic curve CI confidence interval

**Table 3:** Reviewer agreements of markedly hypoechoic, hypoechoic features, and categorization.

Ultrasound features and categorization	Kappa value^a^	Kappa value^b^	*P* value^c^
Markedly hypoechogenicity	0.722 (0.357–1.000)	0.652 (0.411–0.893)	0.753
Hypoechogenicity	0.698 (0.476–0.920)	0.629 (0.397–0.860)	0.673
Categorization	0.705d (0.578–0.832)	0.696e (0.544–0.847)	0.928

**Notes.**

Note: Number in the paragraph is 95% confidence interval.

a Assessment of two reviewers based on original definition and classical C-TIRADS.

b Assessment of two reviewers based on redefinition and modified C-TIRADS.

c Comparison of Kappa values were tested with Z test. d and e:Linear weighted Kappa test.

**Table 4:** Distribution of malignant rates of thyroid nodules using different TIRADS.

	Category	Number of nodule	Benign nodule	Malignant nodule	Malignant rate (%)	Recommended probability (%)
Classical C-TIRADS	2	25	25	0	0.0	0
	3	768	761	7	0.9	<2
	4a	352	267	85	24.1	2–10
	4b	203	66	137	67.5	10–50
	4c	349	41	308	88.3	50–90
	5	24	4	20	83.3	>90
Modified C-TIRADS	2	24	24	0	0.0	0
	3	755	749	6	0.8	<2
	4a	301	265	36	12.0	2–10
	4b	165	65	100	60.6	10–50
	4c	387	55	332	85.8	50–90
	5	89	6	83	93.3	>90
K-TIRADS	2	143	143	0	0.0	<3
	3	722	711	11	1.5	3–10
	4	455	254	201	44.2	10–40
	5	401	56	345	86.0	>60

## Discussion

Interobserver agreement is a crucial indicator of the reproducibility of ultrasound-based malignancy risk stratification. In this study, both the original and redefined criteria for marked hypoechogenicity demonstrated substantial agreement (κ: 0.652–0.722), which is consistent with previous findings, though the redefined criterion achieved a higher kappa value than the classical one (Liu et al., 2023). This improvement can be attributed to the challenge of distinguishing hypoechoic patterns when the thyroid parenchyma exhibits iso- or hyperechoic textures, such as in chronic thyroiditis. In such scenarios, the echogenicity of thyroid nodules may match or closely resemble that of the cervical strap muscles while remaining markedly lower than that of the thyroid parenchyma. These results underscore the importance of standardized echogenicity references in the implementation of TIRADS.

In the present study, the five malignancy-suspicious ultrasound features demonstrated high specificity (92.6–97.4%) but suboptimal sensitivity (15.1–74.5%), particularly under the classical criteria. As shown in Fig. 3, a thyroid nodule with echogenicity similar to the cervical strap muscle was classified as classical C-TIRADS 4b using the original criterion, while the redefined criterion upgraded it to modified C-TIRADS 4c. In contrast, Fig. 5 illustrates a nodule with echogenicity markedly lower than the strap muscle, which was consistently categorized as classical and modified C-TIRADS 5 under both definitions. The redefined marked hypoechogenicity significantly improved sensitivity (74.5% *vs.* 15.1%) and AUC (0.836 *vs.* 0.563), albeit with a modest reduction in specificity (92.6% *vs.* 97.4%). This trade-off aligns with the principle of balanced diagnostic thresholds, wherein enhanced sensitivity facilitates early malignancy detection without critically compromising specificity (Lee et al., 2020; Lee et al., 2022; Delfim et al., 2023). The observed improvement in AUC reflects the inclusion of malignant nodules with echogenicity approximating that of the cervical strap muscles, which were previously misclassified under the stricter original criterion. These findings are consistent with previous studies reporting that the malignancy risk of markedly and moderately hypoechoic nodules is significantly higher than that of mildly hypoechoic nodules (Lee et al., 2020; Lee et al., 2022). Another study also noted similar malignancy probabilities for moderately (38.6%) and markedly (35.4%) hypoechoic nodules, which were slightly higher than that of mildly hypoechoic nodules (26.0%) (Lee et al., 2022).

**Table 5:** Distributions of categories and efficacies of three TIRADS versions for the thyroid nodules.

	Category	Sensitivity (%)	Specificity (%)
Classical C-TIRADS	3	100	2.1
	4a	98.7	67.5
	4b	83.5	90.5
	4c	58.5	96.1
	5	3.6	99.7
Modified C-TIRADS	3	100	2.1
	4a	98.9	66.4
	4b	92.5	89.2
	4c	74.5	94.8
	5	14.9	99.5
K-TIRADS	3	100	12.3
	4	98.0	73.4
	5	61.9	95.2

**Table 6:** Diagnostic efficacy of three TIRADS for the stratification of malignancy risk of thyroid nodules.

	Sensitivity (%)	Specificity (%)	PPV (%)	NPV (%)	Accuracy (%)	AUC (95%)
Classical C-TIRADS	83.5	90.5	80.7	92.0	88.2	0.870^b^ (0.853–0.885)
Modified C-TIRADS	92.5	89.2	80.3	96.1	90.2	0.908^c^ (0.894–0.921)
K-TIRADS	98.0	73.4	63.8	98.7	81.3	0.857^a^ (0.840-0.873)

**Notes.**

Note PPV Positive predictive value NPV Negative predictive value AUC Area under the receiver operating characteristic curve CI confidence interval

a K-TIRADS *vs.* classical C-TIRADS: *P* = 0.169.

b Classical C-TIRADS *vs.* modified C-TIRADS: *P* < 0.001.

c K-TIRADS *vs.* modified C-TIRADS: *P* < 0.001.

The probability of all five malignancy-suspicious ultrasound features occurring simultaneously in a single nodule is low, which explains the small number and proportion of nodules categorized as classical and modified C-TIRADS 5 under both original and modified criteria. This phenomenon has also been observed in other studies, where a considerable proportion of malignant nodules had total scores below five points, leading to an optimal diagnostic cutoff at category 4b or 4c (Chen, Lin & Wu, 2022; Qi et al., 2021; Cai et al., 2023a; Cai et al., 2023b; Si et al., 2024).

A key advantage of the modified C-TIRADS observed in this study is its improved alignment with the malignancy risk ranges recommended in the (classical) C-TIRADS 2020 guidelines. Specifically, the malignancy rate in category 4a decreased significantly and fell within an appropriate range closer to the guideline benchmark, while other categories also showed refined stratification. Compared to the classical C-TIRADS, the modified version demonstrated the following malignancy rates: 12.0% *vs.* 24.1% in category 4a, 60.6% *vs.* 67.5% in category 4b, 85.8% *vs.* 88.3% in category 4c, and 93.3% *vs.* 83.3% in category 5.

The distribution of malignant nodules also differed notably between the two systems. Under the classical C-TIRADS, the malignancy rate in category 4a was 15.3% (85/557), compared to 6.5% (36/557) with the modified system. Conversely, in category 5, the malignancy rate increased from 3.6% (20/557) with the classical system to 14.9% (83/557) with the modified C-TIRADS.

In summary, the substantial decrease in malignancy rate for category 4a nodules reduces the need for fine-needle aspiration or core needle biopsy in this group, while the pronounced increase in malignancy rate for category 5 nodules highlights the diagnostic advantage and clinical optimization offered by the redefined marked hypoechogenicity criterion and the modified C-TIRADS.

Similarly, the malignancy rates of thyroid nodules classified by K-TIRADS 2021 aligned closely with the risk ranges suggested in its guidelines. This finding further supports the validity of the modified C-TIRADS, as different TIRADS applied to the same cohort yielded consistent stratification trends.

Compared to the classical C-TIRADS, the modified version significantly improved sensitivity in detecting malignant nodules, particularly in categories 4 and 5 (74.5% *vs.* 58.5% and 14.9% *vs.* 3.6%, respectively). Overall sensitivity (92.5% *vs.* 83.5%), AUC (0.961 *vs.* 0.920), and NPV were also markedly enhanced. The modified C-TIRADS also demonstrated superior stratification performance over K-TIRADS, with higher specificity (89.2% *vs.* 73.4%), PPV (80.3% *vs.* 63.8%), accuracy (90.2% *vs.* 81.2%), and AUC (0.908 *vs.* 0.857). These results were consistent with those reported by Liu et al. (2023). These findings confirm that the modified C-TIRADS offers significantly improved risk stratification compared to both the classical C-TIRADS and K-TIRADS.

Comet tail artifacts, traditionally regarded as benign indicators (Zhou et al., 2020; Liu et al., 2023), showed no significant association with malignancy in our cohort. Among the 35 nodules exhibiting this feature (20 nodular goiters, two Hashimoto’s thyroiditis, three follicular nodules, and none in thyroid cancer), only one solid hypoechoic nodule with angular margins and vertical orientation was classified as category 4b by classical C-TIRADS and 4c by the modified system. The remaining nodules were categorized as 4a or 3. Using modified C-TIRADS category ≥4b as the malignancy threshold, all 35 nodules were correctly stratified as benign. This aligns with a multicenter retrospective study by Yang et al. (2022), which found no significant difference in comet tail artifact distribution between follicular adenomas and carcinomas.

It should be noted that our study included only histopathologically confirmed nodules. Since comet tail artifacts are commonly observed in colloid cysts or partially liquefied nodules, which are seldom biopsied or resected, leading to such cases were underrepresented in our sample. This may have limited the statistical power to assess the diagnostic value of this feature.

Hashimoto’s thyroiditis and subacute thyroiditis can manifest with diverse sonographic patterns, some of which form “pseudo-nodules” that mimic malignant thyroid nodules (Alexander et al., 2020; Zacharia et al., 2002). In this study, six cases of subacute thyroiditis were included in the benign cohort. Despite their benign nature, all exhibited suspicious ultrasound features, such as solid composition, irregular margins, and hypoechogenicity, resulting in their classification as classical and modified C-TIRADS category 4b or 4c.

This study has several limitations. First, the sample was derived from a single tertiary hospital, and the exclusion of purely cystic and spongiform nodules may have affected the generalizability of the K-TIRADS performance. Second, the use of multiple ultrasound systems and different radiologists introduced inherent interobserver variability. Third, the retrospective design limited the ability to assess dynamic ultrasound features prospectively, which could influence risk stratification accuracy. Finally, the absence of internal and external validation may affect the broader applicability of our findings. Nonetheless, we believe these limitations do not fundamentally undermine the primary conclusions, and we anticipate that in a larger, prospective sample, many of these confounding factors would balance out.

## Conclusions

The adoption of the redefined marked hypoechogenicity criterion in modified C-TIRADS improves diagnostic sensitivity and accuracy in thyroid nodule risk stratification. The modified C-TIRADS optimizes category-specific malignancy rates, particularly by reducing the malignancy probability in category 4a, thereby helping to avoid unnecessary biopsies. Using a cutoff of category ≥4b, the modified system achieved an accuracy of 90.2% and an AUC of 0.908, outperforming both the classical C-TIRADS and K-TIRADS. These improvements support the integration of the modified C-TIRADS into clinical practice for more standardized and efficient thyroid nodule management. The clinical value of the redefined marked hypoechogenicity will be further evaluated in a future prospective study.

##  Supplemental Information

10.7717/peerj.20817/supp-1 Supplemental Information 1 Raw data of thyroids and C-TIRADS and Modified C-TIRADS

10.7717/peerj.20817/supp-2 Supplemental Information 2 Raw data of thyroids and K-TIRADS

10.7717/peerj.20817/supp-3 Supplemental Information 3 STARD checklist

## Additional Information and Declarations

### Data Availability

The following information was supplied regarding data availability: The raw data is available in the Supplemental Files.
